# Anti-Inflammatory, Antioxidant, and Hypolipidemic Effects of Mixed Nuts in Atherogenic Diet-Fed Rats

**DOI:** 10.3390/molecules23123126

**Published:** 2018-11-29

**Authors:** Mee Young Hong, Shauna Groven, Amanda Marx, Caitlin Rasmussen, Joshua Beidler

**Affiliations:** School of Exercise and Nutritional Sciences, San Diego State University, San Diego, CA 92182, USA; shauna.l.groven@eagles.oc.edu (S.G.); amandamarx27@gmail.com (A.M.); caitiecorwin@gmail.com (C.R.); beidler@gmail.com (J.B.)

**Keywords:** nuts, pistachio, lipid profile, antioxidant, oxidative stress

## Abstract

Nut consumption is associated with reduced risk of cardiovascular disease (CVD). Because most studies have administered single nut varieties, it is unknown whether mixed nuts will also reduce CVD risk. The objective of this study was to compare the effects of mixed nut and pistachio consumption on lipid profiles, glucose, inflammation, oxidative stress, and antioxidant capacity in rats fed an atherogenic diet. Thirty male Sprague-Dawley rats (21 days old) were assigned into three groups (*n* = 10) based on initial body weight and fed either an isocaloric control diet (no nuts), 8.1% pistachio diet (single nut), or 7.5% mixed nut diet (almonds, brazil nuts, cashews, macadamia nuts, peanuts, pecans, pistachios, and walnuts) for 8 weeks. Both pistachios and mixed nuts significantly decreased triglycerides, total cholesterol, and LDL-cholesterol (*p* < 0.05) compared with controls. Both nut groups exhibited reductions in C-reactive protein (*p* = 0.045) and oxidative stress (*p* = 0.004). The mixed nut group had greater superoxide dismutase (*p* = 0.004) and catalase (*p* = 0.044) and lower aspartate aminotransferase (*p* = 0.048) activities. Gene expression for *Fas*, *Hmgcr,* and *Cox2* was downregulated for both nut groups compared to controls (*p* < 0.05). In conclusion, mixed nuts and individual nut varieties have comparable effects on CVD risk factors in rats.

## 1. Introduction

Cardiovascular disease (CVD) is the leading cause of mortality in the United States, accounting for 27.9% of all deaths in 2016 [1]. In epidemiological studies, nut consumption is associated with a lower risk of CVD [2,3], diabetes [4], and the metabolic syndrome [5]. As hyperlipidemia is a major CVD risk factor, the benefits of nuts may occur partly through an improved blood lipid profile. Most nuts are low in saturated fatty acids (SFA) but rich in monounsaturated fatty acids (MUFA) and polyunsaturated fatty acids (PUFA) [6]. Replacing SFAs with unsaturated fats has been shown to improve the lipid profile by decreasing triglycerides, total cholesterol, and low-density lipoprotein (LDL) cholesterol [6]. In addition, MUFA may reduce atherosclerosis by inhibiting LDL oxidation [7]. Hargrove et al. reported that peanuts and olive oil, both rich in MUFA, were similarly effective at reducing LDL oxidation in healthy adults [7].

Other beneficial components of nuts include soluble fiber, which lowers serum total and LDL cholesterol by reducing absorption of cholesterol and bile acids in the small intestine [8]. Nuts are rich in some vitamins and minerals that play a role in cardiovascular health, particularly folate, magnesium, and potassium [9]. Folate is important due to its role in homocysteine metabolism [10]. Like potassium, magnesium lowers blood pressure, and it is also required for the function of many enzyme systems related to glucose and lipid metabolism [11]. Finally, both peanuts and tree nuts contain bioactive compounds that may reduce cardiovascular risk, including ellagic acid, flavonoids, and luteolin [12]. For example, Bansode et al. demonstrated that polyphenol-rich peanut skin extract decreased plasma triglyceride and very-low-density lipoprotein (VLDL) levels in rats after 5 weeks [13].

In addition to improving lipid profiles, nut consumption has been linked to lower blood glucose levels [14,15,16,17,18,19]. For example, in women with Type II diabetes, consumption of five or more servings of nuts per week was associated with significantly lower glycemic loads and a 44% reduction in CVD risk [20]. The unsaturated fat, protein, and fiber content of nuts may contribute to their hypoglycemic effects. Many nuts provide significant amounts of magnesium, which improves insulin sensitivity [11], as well as the antioxidants vitamin E and selenium [21,22]. Because oxidative stress plays a crucial role in the pathogenesis of both CVD and diabetes, increased antioxidant intake may ameliorate the risk of these diseases [23]. Bioactive compounds found in nuts may also improve insulin regulation. For example, supplementation with resveratrol, a polyphenol found in peanuts and pistachios, significantly lowered levels of fasting insulin and insulin resistance in patients with Type II diabetes [24].

Although nuts are typically consumed as a mixture in the US, most studies have tested only a single nut variety [25]. Several studies which did use mixed nuts demonstrated improvements in serum lipids and other CVD risk factors [2,19,26]. However, to our knowledge no study has directly compared the effects of mixed nuts and a single nut variety on CVD risk factors in humans. Differences in basal diet and other methodological aspects make it difficult to draw comparisons across studies. Therefore, the objective of this study was to compare the effects of mixed nut and pistachio consumption on lipid profiles, inflammation, oxidative stress, and antioxidant capacity in rats fed an atherogenic diet. Our hypothesis was that mixed nut consumption would reduce the risk for CVD by improving lipid profiles, reducing inflammation and oxidative stress, and increasing antioxidant capacity through favorable modulation of gene expression involving lipid metabolism and inflammation. The direct comparison of mixed nuts with a single nut variety provides a novel aspect to this study. In addition, previous mixed nut studies have used only a few varieties of nuts, whereas our study used a mixture of eight nut varieties, which better reflects how nuts are typically consumed.

## 2. Results

### 2.1. Effect of Nuts on Food and Water Intake and Body Weight

Initial body weight, final body weight, weight gain, daily food intake, and daily water intake were not statistically different among the mixed nut, pistachio, and control groups, as shown in Table 1. The three groups also showed no significant differences in liver, spleen, or epididymal fat weight.

### 2.2. Effect of Nuts on Serum Lipids and Adiponectin

**Figure 1:**
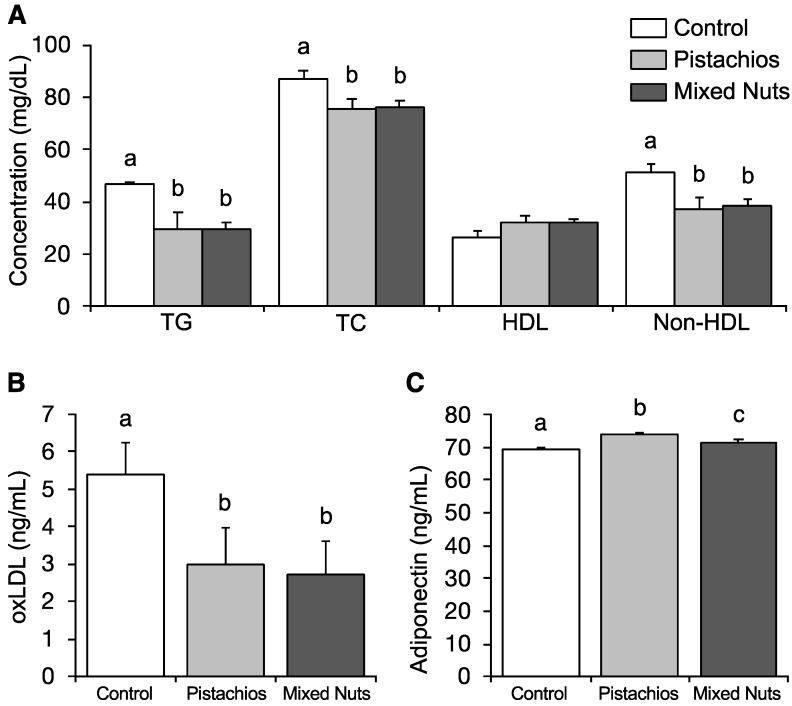
Effects of pistachios and mixed nuts on (**A**) serum lipids, (**B**) oxLDL, and (**C**) adiponectin. Bars represent means ± SE (standard error). Bars with different superscripts differ significantly at *p* < 0.05. TG, triglycerides; TC, total cholesterol; HDL, high-density lipoprotein; oxLDL, oxidized low-density lipoprotein.

Compared with the control group, both the pistachio and mixed nut groups showed significant decreases in triglycerides (*p* = 0.023), total cholesterol (*p* = 0.037), and non-HDL (high-density lipoprotein) cholesterol (*p* = 0.010), as shown in Figure 1A. There were no significant differences in HDL cholesterol among the groups. LDL oxidation is a key step in the formation of atherosclerotic plaques [27]. Circulating oxidized LDL (oxLDL) is a biomarker for CVD risk, and also appears to promote the atherogenic process through a variety of mechanisms [28]. Both the pistachio and mixed nut groups showed a significant reduction in concentrations of oxLDL compared with the control group (*p* = 0.044), as shown in Figure 1B. Adiponectin is a hormone secreted by adipose tissue that promotes glucose utilization and fatty acid oxidation [29]. Both treatment groups showed significantly higher adiponectin levels, and the highest levels were found in the pistachio group (*p* < 0.01), as shown in Figure 1C.

### 2.3. Effect of Nuts on Serum CRP, HMGB1, and Oxidative Stress

**Figure 2:**
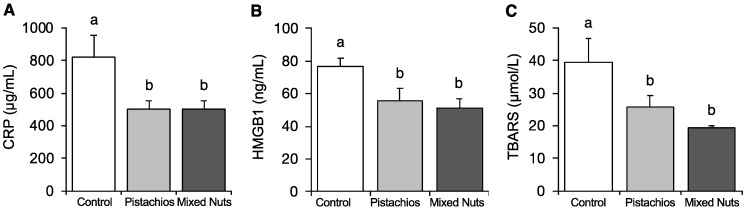
Effects of pistachios and mixed nuts on (**A**) CRP, (**B**) HMGB1, and (**C**) TBARS. Bars represent means ± SE (standard error). Bars with different superscripts differ significantly at *p* < 0.05. CRP, C-reactive protein; HMGB1, high mobility group box 1 protein; TBARS, thiobarbituric acid reactive substances.

C-reactive protein (CRP) and high mobility group box 1 protein (HMGB1) levels were significantly lower in both the pistachio and mixed nut groups compared with the control group (*p* < 0.05), as shown in Figure 2A,B. CRP is a marker for systemic inflammation that significantly predicts cardiovascular events in both men and women [30,31]. HMGB1, a protein released from cells during apoptosis and cytokine stimulation, increases systemic inflammation by binding to various receptors [32]. Compared with the control group, both the pistachio and mixed nut groups showed a significant reduction in thiobarbituric acid reactive substances (TBARS), an indicator of oxidative stress (*p* = 0.004), as shown in Figure 2C [33]. There were no significant differences in CRP, HMGB1, or TBARS between rats in the pistachio and mixed nut groups.

### 2.4. Effect of Nuts on Serum Antioxidant Enzyme Activities

**Figure 3:**
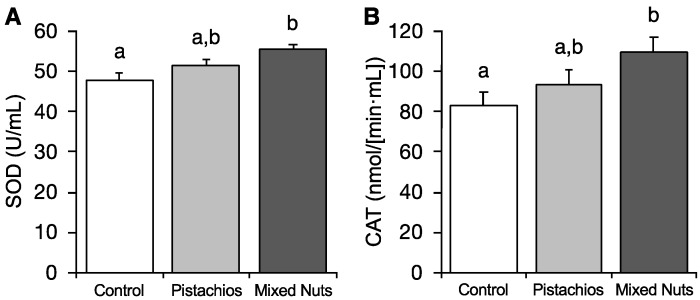
Effects of pistachios and mixed nuts on (**A**) SOD and (**B**) CAT. Bars represent means ± SE (standard error). Bars with different superscripts differ significantly at *p* < 0.05. SOD, superoxide dismutase; CAT, catalase.

Activities of the antioxidant enzymes superoxide dismutase (SOD) (*p* = 0.004) and catalase (CAT) (*p* = 0.044) were significantly higher in the mixed nut group than in control animals, as shown in Figure 3. SOD and CAT activities for the pistachio group were not statistically different from the control or mixed nut groups. No significant differences were detected for glutathione S-transferase (GST), glutathione peroxidase (GPx), or glutathione reductase (GR) among groups.

### 2.5. Effect of Nuts on Serum Liver Function Enzyme Activities

Elevated liver function markers can indicate liver dysfunction associated with a high-fat diet [34]. The mixed nut group showed a significant reduction in serum aspartate aminotransferase (AST) compared with controls (*p* = 0.048), as shown in Table 2. AST for the pistachio group was not statistically different from the control or mixed nut groups. No significant differences were shown for alanine aminotransferase (ALT), alkaline phosphatase (ALP), lactate dehydrogenase (LDH), or creatine kinase (CK).

### 2.6. Effect of Nuts on Hepatic Gene Expression

**Figure 4:**
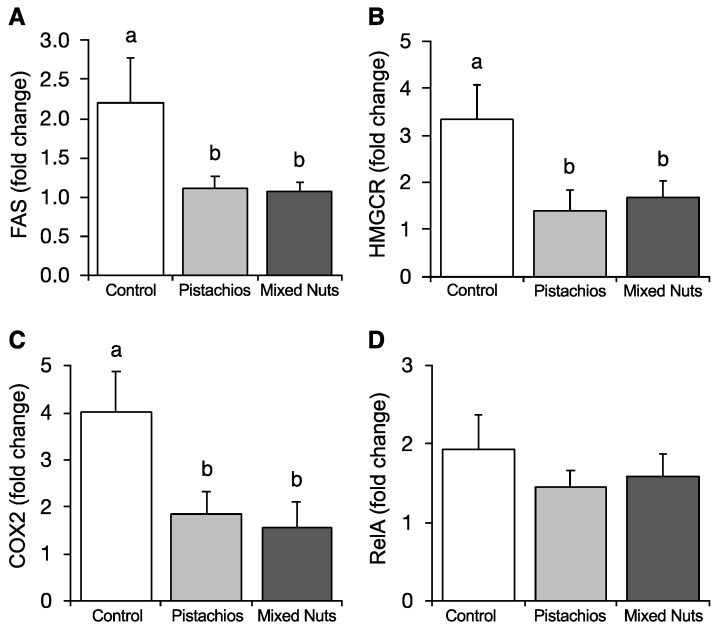
Effects of pistachios and mixed nuts on (**A**) FAS, (**B**) HMGCR, (**C**) COX2, and (**D**) RelA. Bars represent means ± SE (standard error). Bars with different superscripts differ significantly at *p* < 0.05. FAS, fatty acid synthase; HMGCR, 3-hydroxy-3-methylglutaryl-CoA reductase; COX2, cyclooxygenase 2; RelA, nuclear factor NF-kappa-B p65 subunit.

Compared with the control diet, both the pistachio and mixed nut groups downregulated hepatic expression of fatty acid synthase (FAS) (*p* = 0.045), 3-hydroxy-3-methylglutaryl-CoA reductase (HMGCR) (*p* = 0.034), and cyclooxygenase 2 (COX2) (*p* = 0.024), as shown in Figure 4. No significant differences were observed among the groups for nuclear factor NF-kappa-B p65 subunit (RelA).

## 3. Discussion

The objective of this study was to determine whether mixed nuts are similarly effective as a single nut variety for reducing cardiovascular risk factors in rats fed an atherogenic diet. The results suggest that the consumption of mixed nuts is an equally effective approach to reducing cardiovascular risk factors as the consumption of an individual nut variety—pistachios. Both the mixed nut and pistachio groups exhibited an improved lipid profile, as indicated by significant reductions in triglycerides, total cholesterol, and non-HDL cholesterol compared with the control group. In contrast, serum HDL cholesterol was not significantly different among groups. Blood lipids did not differ significantly between the mixed nut and pistachio groups, indicating that these two interventions had similar effects on the lipid profile. Neither intervention produced significant changes in body weight or fat mass, indicating that nuts can improve lipid profile in rats without altering body weight. Similarly, some human studies involving nuts have shown improved lipid profile without changes in anthropometric measurements [35,36,37,38]. Although the effects of mixed nuts on blood lipids have not previously been investigated in animal models, several human trials have shown beneficial results. In the PREDIMED trial, a Mediterranean diet supplemented with 30 g per day of mixed almonds, hazelnuts, and walnuts resulted in a lower total cholesterol (TC)/HDL ratio than the control low-fat diet [19]. Also, in a study of Korean women with metabolic syndrome, the addition of 30 g per day of mixed pine nuts, peanuts, and walnuts lowered total cholesterol and raised HDL cholesterol compared with a healthy control diet [26].

Oxidized LDL concentration was significantly lower in the mixed nut and pistachio groups than in controls. Circulating oxLDL is a known biomarker for atherogenesis, and it may directly promote atherogenesis by increasing oxidative stress through the activation of the NF-κB pathway [28]. In the PREDIMED trial, a Mediterranean diet that included mixed nuts resulted in lower oxLDL than the low-fat control diet [39]. Pistachio consumption has also been associated with lower blood levels of oxLDL in several human trials [14,40].

Serum adiponectin was highest in the pistachio group and higher in the mixed nut group than in controls. Adiponectin, a hormone secreted by adipose tissue, increases glucose uptake and fatty acid oxidation by activating the 5′-AMP-activated protein kinase (AMPK) pathway [29]. Upregulation of AMPK by adiponectin could therefore help to explain the lower levels of triglycerides in the pistachio and mixed nut groups. However, previously published studies have not assessed the effect of pistachios or mixed nuts on adiponectin in animals, and human studies have shown mixed results. For example, Lee et al. found no relationship between mixed nut consumption and adiponectin levels in Korean women with metabolic syndrome [26]. In contrast, Gulati et al. found higher adiponectin levels as well as lower levels of blood glucose and total and LDL cholesterol in Asian Indians with metabolic syndrome [16].

Both pistachio and mixed nut groups exhibited reduced hepatic expression of the genes FAS and HMGCR, which play important roles in lipid synthesis. FAS catalyzes the production of palmitate (C16:0), which is subsequently elongated and desaturated to form other fatty acids [41]. Reduced FAS activity is a possible mechanism for the lower triglyceride levels in the pistachio and mixed nut groups. HMGCR is the rate-limiting enzyme in the synthesis of cholesterol from acetyl-CoA [42]. Reduced HMGCR activity could help explain the lower level of non-HDL cholesterol in the pistachio and mixed nut groups. Previous studies have not evaluated lipid-related gene expression following nut consumption.

Oxidative stress is a key factor in the pathogenesis of atherosclerosis, particularly via the oxidation of LDL cholesterol [43]. TBARS, a biomarker of oxidative load, was significantly lower in the pistachio and mixed nut groups than in the control group. Additionally, serum concentrations of the antioxidant enzymes SOD and CAT were higher in the mixed nut group than in controls. Compared with palm oil, mixed nuts resulted in greater hepatic expression of paraoxonase 2 (Pon2) in ApoE-knockout mice [44]. In the PREDIMED trial, mixed nuts were similarly effective as olive oil for decreasing blood malondialdehyde (MDA) levels [39]. However, in the study by Lee et al., mixed nut consumption did not affect MDA levels in Korean women with metabolic syndrome [26]. Pistachio consumption has also been associated with reduced oxidative stress and increased antioxidant levels. In a study by Alturfan et al., pistachios reduced TBARS and increased total antioxidant capacity in rats fed a high-fat, high-cholesterol diet, but not in rats fed a lower-fat control diet [45]. In separate trials, healthy adults who substituted pistachios for 20% of caloric intake showed decreased oxidative stress, as measured by MDA, and increased indicators of antioxidant status, including SOD [18,46].

Nut consumption has shown the ability to reduce inflammation, a crucial factor in the pathogenesis of cardiovascular disease [47]. In the current study, serum CRP and HMGB1 were significantly lower in the pistachio and mixed nut groups than in the control group. To our knowledge, this study is the first to determine the effects of nut consumption on HMGB1 levels. CRP is a highly sensitive biomarker of systemic inflammation and an important indicator of cardiovascular risk [48]. Gulati et al. reported a significant reduction in CRP following pistachio consumption in adults with metabolic syndrome [16]. HMGB1, a member of the high mobility group family of proteins, is involved in various inflammatory processes [49]. Although primarily located in cell nuclei, HMGB1 is released from cells through the action of proinflammatory cytokines, including tumor necrosis factor-α and interleukin-1. In turn, HMGB1 itself increases production of pro-inflammatory mediators and cytokines, in part by upregulating COX-2 expression [50]. HMGB1 levels are positively correlated with CRP levels in coronary artery disease patients [49].

Both the pistachio and mixed nut groups exhibited reduced hepatic expression of COX-2. This enzyme system, which produces proinflammatory prostaglandins from arachidonic acid, is a therapeutic target of many anti-inflammatory drugs. Reduced expression of COX-2 in the intervention groups may help to explain the reduced inflammation as indicated by lower CRP and HMGB1. Previous studies have not investigated the effect of mixed nuts or pistachios on COX-2 expression in experimental animals or humans. However, several in vitro studies have demonstrated the ability of polyphenol-rich pistachio nut extract to reduce COX-2 expression in murine cell cultures [51,52,53].

Nuts contain a variety of nutrients that may contribute to their cardioprotective effects, including unsaturated fatty acids, fiber, protein, vitamins (vitamin E, riboflavin, and folate), minerals (calcium, magnesium, and potassium), and other bioactive components [54]. Together, these components may reduce CVD risk through a number of mechanisms, including improved lipid profile and endothelial function, reduced inflammation, and lower oxidative stress [55]. A 2015 review of 61 human trials found that nut consumption was associated with significant reductions in TC, triglycerides (TG), and LDL cholesterol, but not HDL cholesterol [56]. These improvements in lipid profile have been attributed to the fact that nuts are rich in monounsaturated fatty acids (MUFAs) and polyunsaturated fatty acids (PUFAs) [6]. PUFAs decrease levels of apo B, the main protein component of LDL, while MUFAs increase levels of apo A1, the major protein component of HDL [21]. Soluble fiber is another component of nuts that lowers cholesterol, possibly by binding bile acids or cholesterol [57]. Reduced oxidation of LDL cholesterol, which was observed in this study, may also contribute to reduced CVD risk. In rats, pistachio consumption significantly increased levels of paraoxonase 1 (PON1) and arylesterase, enzymes that inhibit LDL oxidation [58]. Nut consumption is also associated with increased levels of adiponectin, which promotes fatty acid metabolism while reducing oxidative stress [21]. Vitamins and minerals in nuts may contribute to their cardiovascular health benefits, particularly the B vitamins, vitamin E, and the minerals calcium, magnesium, and potassium [21]. L-arginine, an amino acid found abundantly in nuts, is a precursor of nitric oxide, which promotes vasodilation and also shows the ability to modulate lipid metabolism through effects on cell signaling [59]. Other bioactive compounds found in nuts include proanthocyanidins, flavonoids, and other polyphenols, which function as antioxidants [60]. Some phytochemicals exert their effects through different mechanisms, such as plant sterols, which have been shown to lower plasma TC and LDL concentrations by reducing cholesterol absorption [61]. These various nut components may work synergistically to reduce CVD risk [62].

One limitation of this study stems from the fact that there is no universal definition of mixed nuts. Because nuts vary in their nutritional content, a different combination of nuts than that used in this study may have different effects on measured biomarkers. In addition, because the animals in this study were fed a high-fat atherogenic diet, it is not certain that the same benefits would occur in animals fed a normal diet.

Future studies could investigate the effects of nut consumption in rats fed an overall healthy diet as opposed to the atherogenic diet used in this study. Different dosages of mixed nuts could also be compared to determine the minimum effective dose to improve cardiovascular risk biomarkers. In addition, a study using a longer duration (12 or 16 weeks) may be warranted to evaluate the longer-term effects of nut consumption on CVD risk factors and pathological outcomes. A longer-duration study could look for atherosclerotic lesions to complement the biochemical evidence of cardiovascular risk. Finally, we recommend that future studies compare the quantities of unsaturated fatty acids, polyphenols, and other bioactive compounds in mixed nuts versus a single nut variety.

## 4. Materials and Methods

### 4.1. Animals and Diets

All procedures and training for use of the animals were approved by the San Diego State University Animal Subjects Committee (APF 18-01-001H). Thirty male Sprague-Dawley rats (21 days old) were housed individually in wire-bottomed cages in a research room at San Diego State University on a 12 h light-dark cycle. Both temperature and humidity were controlled at approximately 20–24 °C and 40–45% humidity. Rats were given 3–4 days to acclimate to their environment before the study began. Rats were equally divided into three groups of ten, consuming isocaloric diets consisting of either control diet, 8.1% pistachio diet, or 7.5% mixed nut diet for 8 weeks, as shown in Table 3. Nuts account for approximately 9.8% of total kcals for the pistachio diet or mixed nut diet. Nut dosages were chosen to provide a similar percentage of total kcals to human studies [38]. All ingredients except nuts were purchased from Dyets (Bethlehem, PA, USA). Pistachios were obtained from The Wonderful Company (Lost Hills, CA, USA). Mixed nuts, peanuts, and walnuts were Kirkland brand products from Costco (Issaquah, WA, USA). The mixed nuts contained almonds, brazil nuts, cashews, macadamia nuts, and pecans. Because some commonly consumed nuts were absent from this product, 1.125 g walnuts (15%), 1.125 g pistachios (15%), and 1.5 g peanuts (20%) were added per 3.75 g of the basic mixture (50%). Water and food were available at all times for the animals. Body weight was measured weekly throughout the study, while food and water intake were measured at weeks 3, 5, and 7.

At the conclusion of the study, animals were euthanized by CO_2_ exposure. Blood was collected and centrifuged for 10 min at 1200× *g* at 4 °C. Serum was stored at −80 °C until time of analysis. Liver, spleen, and epididymal fat pads were harvested and weighed.

### 4.2. Serum Lipids and Oxidized LDL

Serum total cholesterol, HDL-cholesterol, and triglycerides were measured using assay kits from Stanbio (Boerne, TX, USA). Non-HDL-cholesterol was calculated by subtracting HDL-cholesterol from total cholesterol; then total triglycerides divided by five was subtracted from the previous number obtained. Oxidized LDL was measured using an oxLDL sandwich ELISA kit from MyBioSource (San Diego, CA, USA). Serum samples were incubated with anti-rat oxLDL antibody, followed by biotinylated detection antibody. Avidin-horseradish peroxidase conjugate was added to produce a blue color in direct proportion to initial oxLDL concentration. Following the addition of a stop solution, absorbance was read at 450 nm.

### 4.3. Serum Adiponectin

Adiponectin was measured using a sandwich ELISA kit from R&D Systems (Minneapolis, MN, USA). Serum samples were incubated with anti-rat adiponectin antibody, followed by horseradish peroxidase-conjugated anti-rat adiponectin antibody. Substrate solution was added to produce a blue color in proportion to the concentration of adiponectin in the original sample. A stop solution was added, and absorbance was read at 450 nm.

### 4.4. Serum C-Reactive Protein

C-reactive protein was measured using a sandwich ELISA kit from BD Biosciences (San Jose, CA, USA). Serum samples were incubated with rabbit anti-rat CRP antibody, followed by horseradish peroxidase-conjugated anti-rat CRP antibody. A 3,3′,5,5′-tetramethylbenzidine substrate solution was used to produce a blue color in direct proportion to initial CRP concentration. Following the addition of a stop solution, absorbance was read at 450 nm.

### 4.5. Serum HMGB1 Activity

High mobility box group 1 protein (HMGB1) was measured using a sandwich ELISA kit from MyBioSource. Serum samples were incubated with anti-rat HMGB1 antibody and horseradish peroxidase-conjugated anti-rat HMGB1 antibody, then substrate solution was added to produce a blue color in direct proportion to initial HMGB1 concentration. The absorbance was read at 450 nm.

### 4.6. Serum Thiobarbituric Acid Reactive Substances

Oxidative stress was measured using a thiobarbituric acid reactive substances (TBARS) assay kit (Cayman, Ann Arbor, MI, USA). Serum samples were combined with sodium docecyl sulfate solution and color reagent and boiled for 1 h, then the reaction was stopped by putting the samples on ice. The samples were stabilized at room temperature before being centrifuged, and absorbance was read at 355 nm.

### 4.7. Serum Antioxidant Enzyme Activities

Serum levels of superoxide dismutase (SOD), catalase (CAT), glutathione peroxidase (GPx), glutathione S-transferase (GST), and glutathione reductase (GR) were determined using assay kits from Cayman. To determine SOD activity, xanthine oxidase and hypoxanthine were added to the samples to catalyze the formation of superoxide radicals, then absorbance was read at 450 nm. To determine CAT activity, the reaction between the enzyme and methanol was determined colorimetrically in the presence of H_2_O_2_. Absorbance was read at 540 nm. GR was determined by measuring the rate of oxidation of NADPH to NADP^+^, which was directly proportional to the GR activity in the sample. GPx activity was measured indirectly through a coupled reaction with GR. Oxidized glutathione (GSSG), produced through the reduction of hydroperoxide by GPx, was subsequently recycled to its reduced state by GR. During this reaction, NADPH was oxidized to NADP^+^, causing a decrease in absorbance directly proportional to GPx activity. GST activity was determined by measuring the conjugation of 1-chloro-2,4-dinitrobenzene with reduced glutathione. This reaction caused an increase in absorbance that was directly proportional to GST activity in the sample. Absorbance for GR, GPx, and GST was read at 340 nm. All antioxidant assays were carried out according to the manufacturer’s instructions.

### 4.8. Serum Liver Function Enzyme Activities

Serum levels of alkaline phosphatase (ALP), alanine aminotransferase (ALT), aspartate aminotransferase (AST), creatine kinase (CK), and lactate dehydrogenase (LDH) were determined using assay kits from Stanbio. Assays were performed according to the manufacturer’s instructions. All enzyme assays were read at 340 nm, except for ALP, which was read at 405 nm.

### 4.9. Hepatic Gene Expression

Following the extraction of total ribonucleic acid (RNA) from liver samples via the TRIzol method, reverse transcription was performed using Superscript III Reverse Transcriptase and oligo(dT)12–18 primers from Invitrogen (Carlsbad, CA, USA). Quantitative real-time polymerase chain reaction (PCR) was performed using the ViiA 7 real-time PCR system and TaqMan Universal PCR probes from Applied Biosystems (Foster City, CA, USA) to determine mRNA levels of fatty acid synthase (FAS), 3-hydroxy-3-methylglutaryl-CoA reductase (HMGCR), cyclooxygenase 2 (COX-2), and nuclear factor NF-kappa-B p65 subunit (RelA). Relative quantitation values of the target genes were calculated using ∆∆CT numerical quantity and normalized to r18S expression. As an additional safeguard against contamination or anomalies, each set of reverse transcription reactions contained a minus reverse transcription negative control.

### 4.10. Statistical Analysis

All data were analyzed by an ANOVA procedure using SPSS (IBM, Armonk, NY, USA) to evaluate the effects of the experimental diets on variables. Post hoc tests were performed using Student–Newman–Keuls (SNK) tests. Data are presented as mean ± SE (standard error), and an alpha level of *p* < 0.05 is considered significant.

## 5. Conclusions

In conclusion, these results suggest that the consumption of mixed nuts is a practical approach to reducing cardiovascular risk factors that is equally effective as the consumption of an individual nut variety, pistachios. Mixed nuts reduced CVD risk by improving the blood lipid profile, including significant reductions in triglycerides, total cholesterol, and non-HDL cholesterol. Mixed nuts also increased adiponectin while significantly reducing the serum concentration of oxidized LDL. Reduced hepatic expression of FAS and HMGCR may have contributed to the improved lipid profile in rats fed the mixed nut diet, while reduced COX-2 expression may help to explain the reductions in the serum inflammation markers CRP and HMGB1. Oxidative stress was also reduced in the mixed nut group, as indicated by lower TBARS and higher activities of CAT and SOD.

## Figures and Tables

**Table 1:** Body and organ weights and food and water intake of rats.

	Control	Pistachios	Mixed Nuts
Initial body weight (g)	49.29 ± 1.11	49.77 ± 0.88	49.85 ± 0.84
Final body weight (g)	369.4 ± 4.49	377.6 ± 4.95	378.3 ± 7.86
Weight gain (g)	320.1 ± 5.08	327.8 ± 4.37	328.4 ± 7.61
Food intake (g/d)	18.21 ± 0.46	18.79 ± 0.40	20.02 ± 0.65
Water intake (g/d)	26.18 ± 0.91	27.38 ± 1.28	26.79 ± 1.23
Liver weight (g)	22.11 ± 0.62	21.21 ± 0.86	22.00 ± 0.87
Spleen weight (g)	1.64 ± 0.13	1.59 ± 0.11	1.47 ± 0.17
Epididymal fat weight (g)	3.67 ± 0.14	3.56 ± 0.17	3.41 ± 0.15

Data are presented as means ± SE (standard error). Data were tested using one-way ANOVA followed by a Student–Newman–Keuls (SNK) test for between-group comparisons. n = 30, 10 animals per group.

**Table 2:** Liver function enzyme activities in rats.

	Control	Pistachios	Mixed Nuts
AST (U/L)	40.29 ± 3.94 ^a^	34.51 ± 0.53 ^a,b^	31.00 ± 1.88 ^b^
ALT (U/L)	28.16 ± 1.66	29.86 ± 2.29	33.19 ± 2.41
ALP (U/L)	63.35 ± 2.77	58.78 ± 2.54	64.32 ± 2.50
LDH (U/L)	97.44 ± 9.63	116.9 ± 17.8	108.3 ± 7.19
CK (U/L)	633.2 ± 64.9	600.6 ± 96.7	656.8 ± 57.1

Data are presented as means ± SE. Data were tested using one-way ANOVA followed by a SNK test for between-group comparisons. Data within rows with varying superscript letters are statistically different (*p* < 0.05). *n* = 30, 10 animals per group. ALP, alkaline phosphatase; ALT, alanine aminotransferase; AST, aspartate aminotransferase; CK, creatine kinase; LDH, lactate dehydrogenase.

**Table 3:** Composition of experimental diets.

Ingredient (g)	Control	Pistachios	Mixed Nuts
Cornstarch	14.30	13.40	13.50
Sucrose	33.00	32.40	32.70
Cellulose	5.00	4.10	4.50
Casein	20.00	18.30	18.70
Corn oil	5.00	5.00	5.00
Milk fat	16.00	12.40	11.70
Cholesterol	1.00	1.00	1.00
Salt mix	3.50	3.50	3.50
Vitamin mix	1.00	1.00	1.00
DL-Methionine	0.30	0.30	0.30
Sodium cholate	0.50	0.50	0.50
Choline bitartrate	0.40	0.40	0.40
TBHQ	0.003	0.003	0.003
Pistachios	0.00	8.10	0.00
Mixed nuts	0.00	0.00	7.50
Total	100.00	100.00	100.00

By weight, each diet provided 33% sugar, 21% fat, and 1% cholesterol. TBHQ: tert-butylhydroquinone.
